# Risk for second primary malignancies in patients with multiple myeloma: a systematic review and meta-analysis

**DOI:** 10.3389/fonc.2026.1770120

**Published:** 2026-04-07

**Authors:** Yingjie Tian, Liang Su, Yujin Li, Minlan Ye, Yuchu Zhang, Qiwei Li, Yongzheng Jiao, Jie Wu

**Affiliations:** 1 Guang’anmen Hospital, China Academy of Chinese Medical Sciences, Beijing, China; 2 Graduate School, Beijing University of Chinese Medicine, Beijing, China; 3 The First People’s Hospital of Yunnan Province, The Affiliated Hospital of Kunming University of Science and Technology, Kunming, Yunnan, China

**Keywords:** meta-analysis, multiple myeloma, second primary malignancies, second primary solid tumor, second primary hematologic malignancy

## Abstract

**Background:**

With improving survival in multiple myeloma (MM), second primary malignancies (SPMs) remain an important issue in long-term care. This study assessed the risk of SPMs among individuals with MM.

**Methods:**

We systematically searched EMBASE, PubMed, and the Cochrane Library for studies published up to August 15, 2025, and pooled standardized incidence ratios (SIRs) to compare SPM risks in MM patients with those in the general population.

**Results:**

Of 1602 records screened, 15 studies comprising 279,894 MM patients met the inclusion criteria. The overall risks of SPMs and solid tumors were not significantly higher than those in the general population. In contrast, the risk of hematologic SPMs was markedly increased (SIR = 2.91; 95% CI: 1.57–5.41). Higher risks were identified for several malignancies, including non-Hodgkin lymphoma (NHL), myelodysplastic syndromes (MDS), acute myeloid leukemia (AML), mesothelioma, skin cancer, melanoma, endocrine tumors, and thyroid cancer. Reduced risks were observed for chronic lymphocytic leukemia (CLL), head and neck cancer, tracheal/bronchial/lung cancer, bladder cancer, and breast cancer. Subgroup analyses showed no meaningful variation by diagnostic period, latency, age, or sex.

**Conclusion:**

These findings demonstrate that MM have a distinct SPM pattern, underscoring the need for tumor-specific surveillance with particular attention to hematologic SPMs and selected solid tumors. The lower incidence of breast cancer is an unexpected and potentially informative signal that could help guide future research. Overall, follow-up strategies should be shaped by site-specific risks rather than applied uniformly across all SPMs.

**Systematic Review Registration:**

https://www.crd.york.ac.uk/PROSPERO/, identifier CRD420251087056.

## 1 Introduction

Multiple myeloma (MM) is currently the second most frequent hematologic cancer worldwide, with roughly 188,000 new diagnoses each year (1, 2). Its therapeutic landscape has undergone substantial changes over the past two decades. Wider use of autologous stem cell transplantation (ASCT) and the introduction of immunomodulatory drugs, proteasome inhibitors, monoclonal antibodies, CAR T-cell therapy, and bispecific antibodies have extended survival for many patients (3–5). These gains have also highlighted long-term complications. Among them, second primary malignancies (SPMs) have become a major concern in the long-term care of patients with MM (6–10).

Recognition of SPM risk in MM has evolved over time (11). Reports from the 1970s already described an excess of secondary acute leukemia in patients with MM, particularly among those exposed to alkylating agents such as melphalan (12, 13). Subsequent studies further established the association between alkylator exposure and therapy-related myeloid neoplasms, with myelodysplastic syndromes (MDS) and acute myeloid leukemia (AML) emerging as the predominant hematologic SPMs (14–16). At the same time, accumulating evidence suggests that the increased risk of myeloid malignancies may not be fully explained by treatment exposure alone, as disease-related susceptibility may already be present in MM and even in its precursor state, monoclonal gammopathy of undetermined significance (MGUS), which was associated with an 8.01-fold increased risk of AML/MDS in a Swedish population-based study (7, 17). As treatment strategies evolved, modern regimens incorporating novel agents, ASCT, and CAR T-cell therapy have improved disease control but may also influence SPM risk (6, 18–21). Lenalidomide maintenance has received particular attention. Several phase III trials and meta-analyses have shown that lenalidomide is associated with an increased risk of SPMs (18, 22–24). This risk appears to be further increased when lenalidomide is combined with oral alkylating agents, and has been observed in both transplant and non-transplant settings, involving both hematologic and solid tumor SPMs (15, 18, 25, 26). More recently, the spectrum of hematologic SPMs in MM has been suggested to extend beyond AML and MDS, with acute lymphoblastic leukemia (ALL) emerging as a rare but increasingly recognized event after MM (27). In this context, accumulating evidence has raised concern about a possible association between prolonged lenalidomide exposure, particularly in the maintenance setting, and the development of ALL, which has been described in selected patients after ASCT and appears to be predominantly Philadelphia chromosome-negative (27–32). Whether ASCT itself increases SPM risk remains uncertain. Some large cohort studies have reported higher SPM rates after ASCT; however, most evidence suggests that alkylating agents used in conditioning regimens are the principal contributors to secondary hematologic malignancies (15, 33). In contrast, the proteasome inhibitor bortezomib may be associated with a lower risk of second malignancies (7, 34). SPMs are associated with poor prognosis, and MM patients who develop them face increased mortality, underscoring the importance of this issue in contemporary survivorship care (9, 35–37).

Several reviews, consensus statements, and meta-analyses have examined second primary malignancies (SPMs) in patients with multiple myeloma (MM) (18, 38, 39). However, most previous studies have focused on treatment-specific associations, particularly those related to lenalidomide, anti-CD38 monoclonal antibodies, or cellular therapies, and have generally summarized relative treatment effects using odds ratios, risk ratios, or hazard ratios rather than standardized incidence ratios (SIRs) (18, 38, 39). Because patients with MM are typically exposed to multiple sequential or combined therapies over the course of their disease, treatment-specific estimates may not fully reflect the overall burden of SPMs at the population level. By contrast, SIRs compare the observed incidence of SPMs in MM patients with the incidence expected in the general population, thereby providing a broader estimate of excess risk beyond individual therapeutic exposures. To address this gap, we conducted a meta-analysis to compare the incidence of SPMs in patients with MM with the expected incidence in the general population.

## 2 Methods

### 2.1 Registration

This study was registered with PROSPERO (ID: CRD420251087056).

### 2.2 Literature retrieval

We searched EMBASE, The Cochrane Library, and PubMed from database inception to August 15, 2025. Search terms included “Multiple Myeloma”, “Smoldering Multiple Myeloma”, “Neoplasms, Second Primary”, “Second Primary Neoplasm”, and “Therapy-Associated Neoplasm”. We used both MeSH terms and free text. We also checked reference lists of relevant articles and reviews. The search was limited to studies published in English. The full PubMed strategy is provided in **Supplementary Appendix 1**.

### 2.3 Study selection

We included studies that met all of the following criteria. (1) Patients had a confirmed diagnosis of multiple myeloma. (2) The study reported risk of SPMs in MM patients and compared this risk with that in the general population. (3) The study provided SIR with 95% CI or supplied data that allowed their calculation. (4) The article was an original study published in English with full text available.

We excluded studies for these reasons. (1) Duplicate reports from the same cohort. In such cases we kept the most recent or the most complete report. (2) Conference abstracts, reviews, editorials, letters, case reports, animal studies. (3) Studies for which SIRs and 95% CIs could not be obtained.

Two investigators (Y-j T and LS) screened records independently. They removed duplicates and screened titles and abstracts to exclude clearly irrelevant reports. They then reviewed full texts for eligibility. Disagreements were resolved by discussion with a third reviewer (JW).

### 2.4 Data extraction and quality assessment

Two investigators (Y-j T and LS) extracted data independently and cross checked the results. Extracted items included first author, publication year, study period, database, region, sample size, stratification details, follow-up time, cancer sites, and SIRs with 95% CIs. We used the Newcastle-Ottawa Scale (NOS) to assess methodological quality. Scores of 4–6 were taken to reflect moderate quality, and scores above 6 were judged as high quality (40, 41). Any discrepancies in data extraction or quality scoring were resolved by joint re-examination and consensus.

### 2.5 Statistical analysis

We pooled SIRs and 95% CIs using the random-effects model in Stata version 18.0, as this approach provides more conservative estimates (42). Heterogeneity was assessed with Cochran’s Q test and I^2^. We interpreted I^2^ as follows. 0-25% indicated low heterogeneity. 25-50% indicated moderate heterogeneity. Values>50% indicated substantial heterogeneity (43). Sensitivity analyses were performed using a leave-one-out approach by sequentially omitting individual studies. We performed subgroup analyses by cancer site, year of diagnosis, latency period, age and sex. When more than ten studies were available for an outcome, we assessed publication bias by visual inspection of funnel plots and by Begg’s and Egger’s tests. A P value<0.05 was considered statistically significant (43).

## 3 Results

### 3.1 Literature retrieval

**Figure 1:**
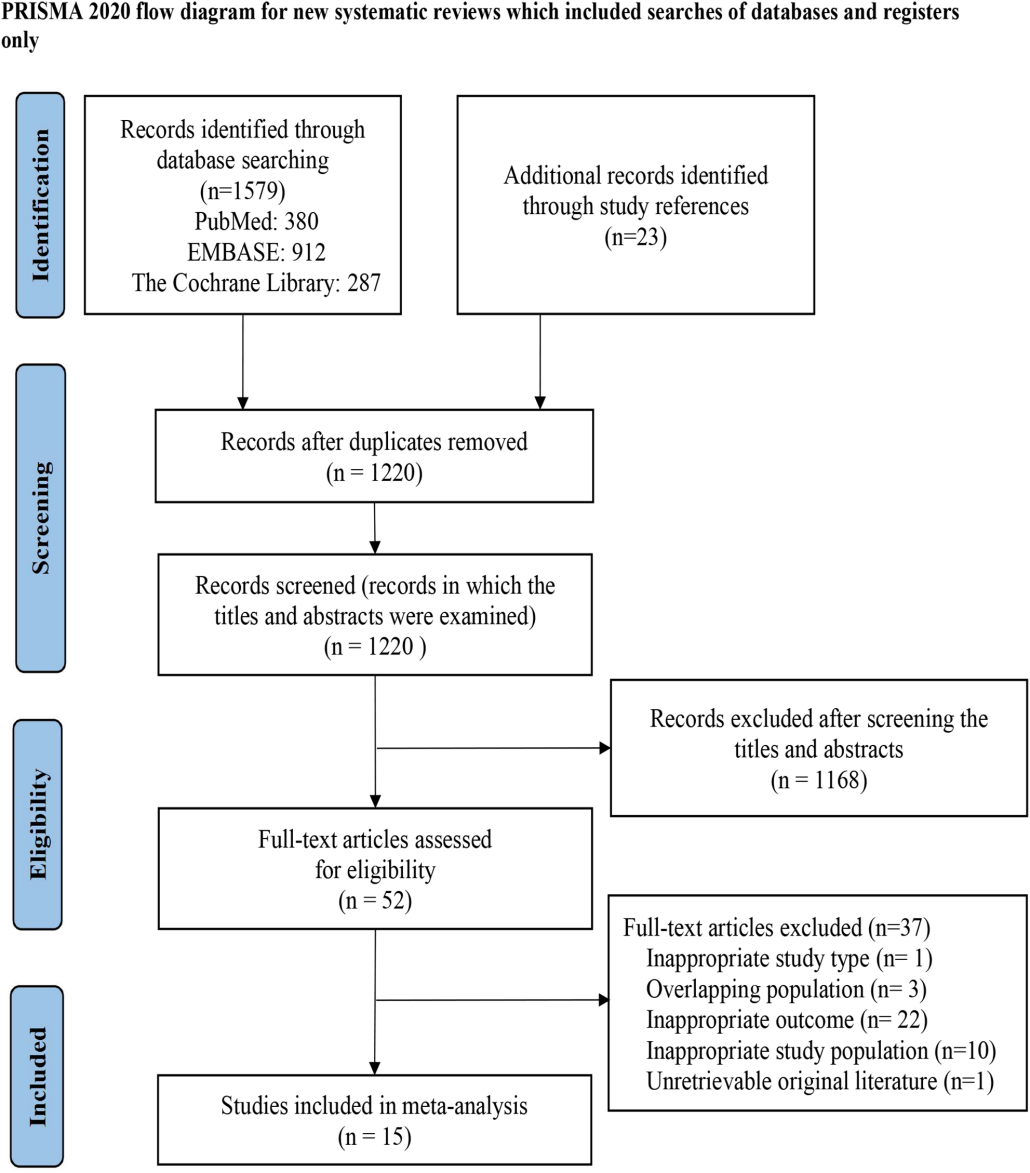
Flow diagram for the study selection process.

The search identified 1602 records from the three databases and reference lists. After removing 382 duplicates, 1220 titles and abstracts were screened, and 1168 records were excluded. We reviewed 52 full-text articles for eligibility. Thirty seven studies were excluded for the following reasons: inappropriate study type (n = 1), overlapping populations (n = 3), inappropriate outcomes (n = 22), inappropriate study population (n = 10), and unavailable original literature (n = 1). A total of 15 studies met all inclusion criteria. The study selection process is shown in **Figure 1**, and additional details are provided in **Supplementary Appendix 2**.

### 3.2 Study characteristics

Fifteen studies were included, and one study reported data from two countries, which were treated as separate cohorts. In total, 279,894 patients were analyzed (17, 44–56). All studies compared the risk of SPMs in MM patients with the expected cancer incidence in the general population. Seven studies were conducted in America (44, 45, 48, 51, 54–56), four in Sweden (17, 49, 52, 53), two in Finland (46, 57), one in Korea (47), one in Spain (50), and one in Germany (53). Two studies received a NOS score of 6 and were rated as moderate quality (50, 56), while the remaining studies were assessed as high quality. Detailed characteristics of the included studies are summarized in **Table 1**.

**Table 1:** Baseline characteristics of the included studies.

Authors	Year	Database	Region	Period	Sample size	Follow-Up (y)	NOS
Mahindra et al. (44)	2014	CIBMTR	America	1990-2010	4161	0.3-16	7
Mailankody et al. (17)	2011	SCR	Sweden	1986 - 2005	8740	27.9*	8
Wang et al. (45)	2022	SEER^a^	America	1975 - 2018	43825	3.7*	8
Rönkkö et al. (46)	2024	FCR	Finland	1992-2019	45533	0.5–5+	8
Park et al. (47)	2023	NHIS-NHID	Korea	2010-2018	9985	2.3**	7
Greene et al. (48)	1985	Connecticut^b^	America	1935-1982	2249	2**	7
Hemminki et al. (49)	2003	FCD^c^	Sweden	1958-1998	65120	0-10+	7
Fernández-Caballero et al. (50)	2019	SMS	Spain	1991-2018	403	3.33*	6
Dong et al. (51)	2025	SEER^a^	America	1992-2020	60550	5*	8
Dong et al. (52)	2001	FCD^c^	Sweden	1958-1996	8656	2.5*	8
Chen et al. (53)	2016	German^d^	Germany	1997-2010	18735	2.6**	7
Chen et al. (53)	2016	FCD^c^	Sweden	1997-2010	7560	2.6**	7
Chakraborty et al. (54)	2012	SEER^a^	America	1973-2008	3245	0.08-20+	8
Stegman et al. (55)	1979	SWOG	America	1965.9-1974.9	628	0.17-12+	7
	2002	FLG^e^	Finland	1979-1985	432	16**	7
Rossi et al. (56)	2013	BiRd cohort^f^	America	2004-2006	72	6.6*	6

*Median follow-up time; ** Mean follow-up time. CIBMTR: the Center for International Blood and Marrow Transplant Research; SCR, the Swedish Cancer Registry; SEER^a^, Surveillance, Epidemiology, and End Results; These three studies all utilized the SEER database; however, we employed non-overlapping datasets, and in cases of overlapping populations, data from the study reporting the largest sample size were selected; FCR, the Finnish Cancer Registry; NHIS-NHID, the national health insurance system - National Health Information Database; Connecticut^b^, The study utilized a cohort of 19,000 persons with initial cancers of the lymphatic and hematopoietic system in Connecticut between 1935 and 1982; FCD^c^, the Swedish Family-Cancer Database, however, we employed non-overlapping datasets, and in cases of overlapping populations, data from the study reporting the largest sample size were selected; SMS, the Servicio Murciano de Salud Healthcare System; German^d^, Latest version of a pooled database from 12 population-based German cancer registries; SWOG, Southwest Oncology Group; FLG^e^, the Finnish Leukemia Group, The 432 patients for this study participated in the four Finnish Leukemia Group (FLG) trials which were open to new myeloma patients from 1979 to 1985; BiRd cohort^f^: A phase II trial of frontline BiRd (clarithromycin, lenalidomide, dexamethasone) in 72 treatment-naive patients with multiple myeloma.

### 3.3 Overall risk of SPMs and subgroup analyses

**Figure 2:**
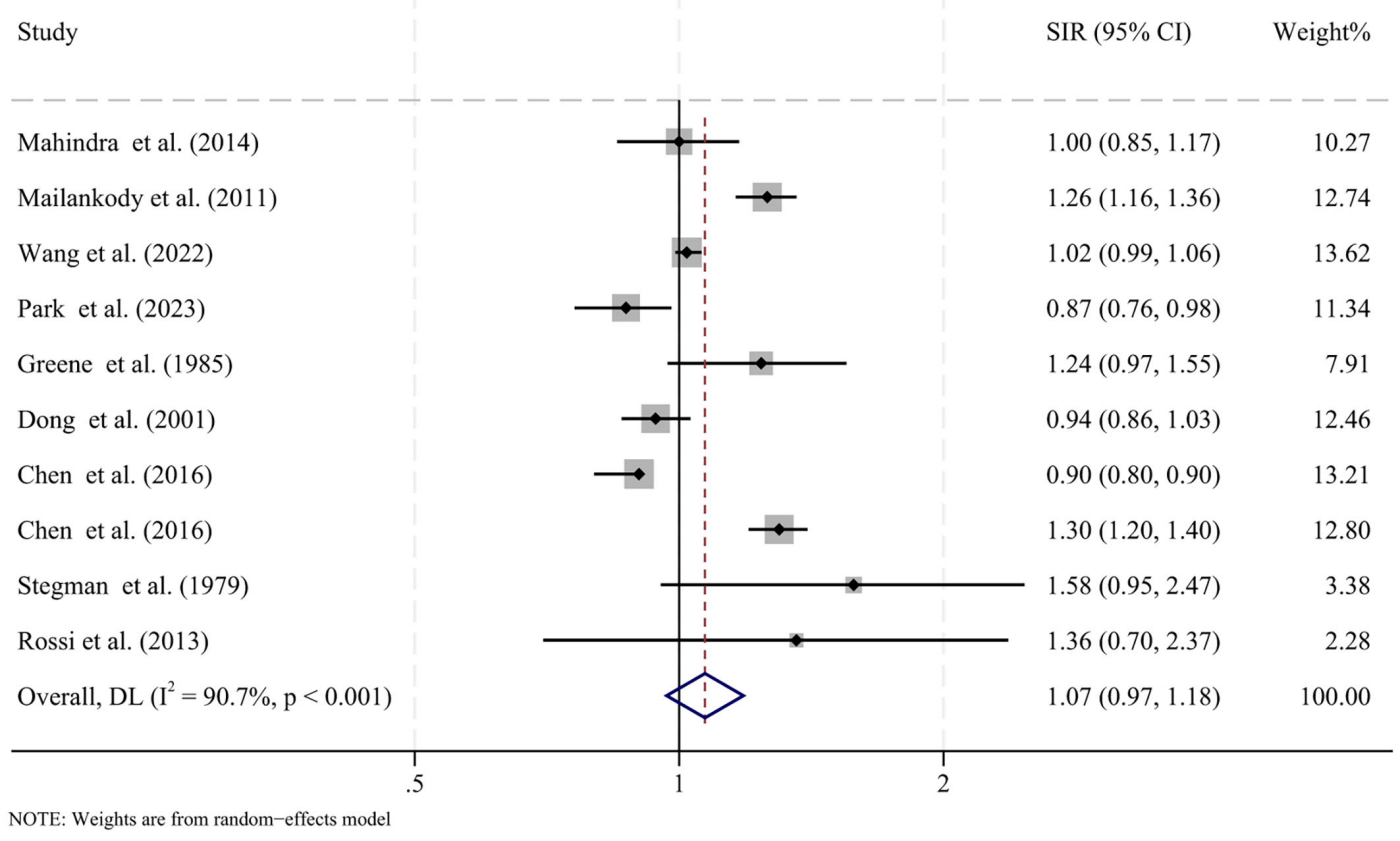
Forest plot showing the pooled standardized incidence ratios (SIRs) for overall second primary malignancies in patients with MM compared with the general population. CI, confidence interval; SIR, standardized incidence ratio; I2, inconsistency.

We analyzed data from 10 studies to evaluate the risk of SPMs in patients with MM. The pooled SIR was 1.07 (95% CI: 0.97-1.18, P = 0.187), showing no significant increase in SPM risk compared to the general population (**Figure 2**). Substantial heterogeneity was observed across studies (I^2^ = 90.7%, P < 0.001). Sensitivity analyses, conducted by excluding one study at a time, yielded consistent results (**Supplementary Figure 1**). We performed subgroup analyses to assess whether diagnosis year (before vs. after 2000), latency period (≤5 years vs. >5 years), age (≤60 years vs. >60 years) at MM diagnosis, or sex influenced the SPM risk (**Table 2**). No significant differences were found between groups.

**Table 2:** Results of the subgroup analyses.

Parameters	N	Association		Heterogeneity		Difference P Value
		SIR (95%CI)	P value	I^2^ (%)	P value	
Year of Diagnosis						
All malignancies						
<2000s	2	1.31 (0.78-2.19)	0.308	99.5	<0.001	0.924
>2000s	2	1.36 (0.80-2.31)	0.262	99.7	<0.001	
Hematologic malignancies						
<2000s	2	2.99 (2.20-4.07)	<0.001	54.8	0.137	0.963
>2000s	2	3.08 (0.96-9.83)	0.058	99.6	<0.001	
Solid tumors						
<2000s	2	1.20 (0.72-2.00)	0.495	99.5	<0.001	0.914
>2000s	2	1.16 (0.79-1.68)	0.451	97.4	<0.001	
Latency Period						
All malignancies						
≤5 years	2	1.24 (0.69-2.22)	0.465	99.7	<0.001	0.694
>5 years	2	1.44 (0.90-2.33)	0.132	99.2	<0.001	
Hematologic malignancies						
≤5 years	2	2.79 (0.82-9.47)	0.100	98.50	<0.001	0.723
>5 years	2	3.56 (1.98-6.41)	<0.001	95.70	<0.001	
Solid tumors						
≤5 years	3	1.03 (0.77-1.37)	0.832	98.4	<0.001	0.261
>5 years	3	1.34 (0.94-1.89)	0.103	95.0	<0.001	
Age						
All malignancies						
≤60 years	2	1.61 (0.89-2.89)	0.113	99.1	<0.001	0.512
>60 years	2	1.24 (0.74-2.07)	0.418	99.8	<0.001	
Hematologic malignancies						
≤60 years	2	4.82 (1.84-12.57)	0.001	98.9	<0.001	0.342
>60 years	2	2.55 (1.04-6.23)	0.040	96.4	<0.001	
Solid tumors						
≤60 years	2	1.32 (0.83-2.09)	0.242	98.9	<0.001	0.597
>60 years	2	1.11 (0.72-1.71)	0.629	98.3	<0.001	

95% CI, 95% Confidence interval; N, Number of cohorts; SIR, Standardized incidence ratio.

### 3.4 Risk of hematologic SPMs and subgroup analyses

**Figure 3:**
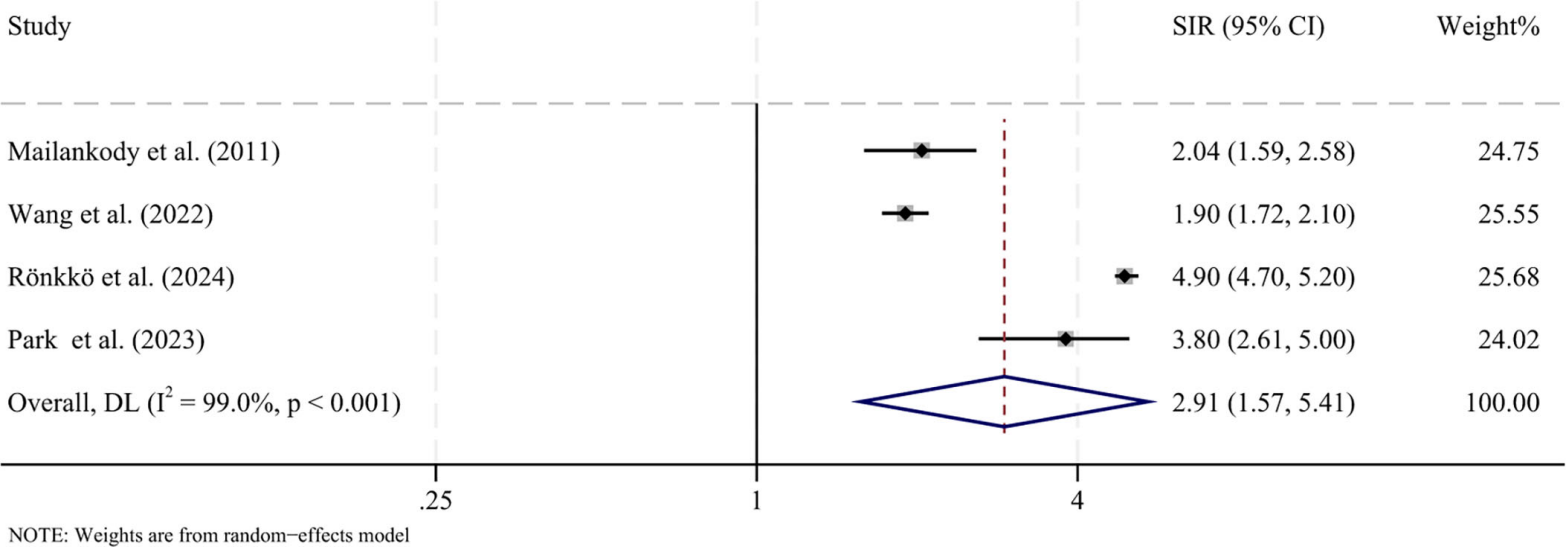
Forest plot presenting the pooled standardized incidence ratios (SIRs) for second primary hematologic malignancies in patients with MM compared with the general population. CI, confidence interval; SIR, standardized incidence ratio; I2, inconsistency.

We conducted stratified analyses by cancer type. Four studies reported on the risk of hematologic malignancies. The pooled SIR was 2.91 (95% CI: 1.57-5.41, P = 0.001), indicating a significantly elevated risk compared with the general population (**Figure 3**). Study heterogeneity was high (I^2^ = 99.0%, P < 0.001). Sensitivity analysis confirmed the robustness of the result (**Supplementary Figure 2**). Subgroup analyses were performed to explore the influence of diagnosis year (before vs. after 2000), latency period (≤5 years vs. >5 years), age (≤60 years vs. >60 years), and sex (**Table 2**). No significant differences were observed across subgroups.

### 3.5 Risk of solid SPMs and subgroup analyses

**Figure 4:**
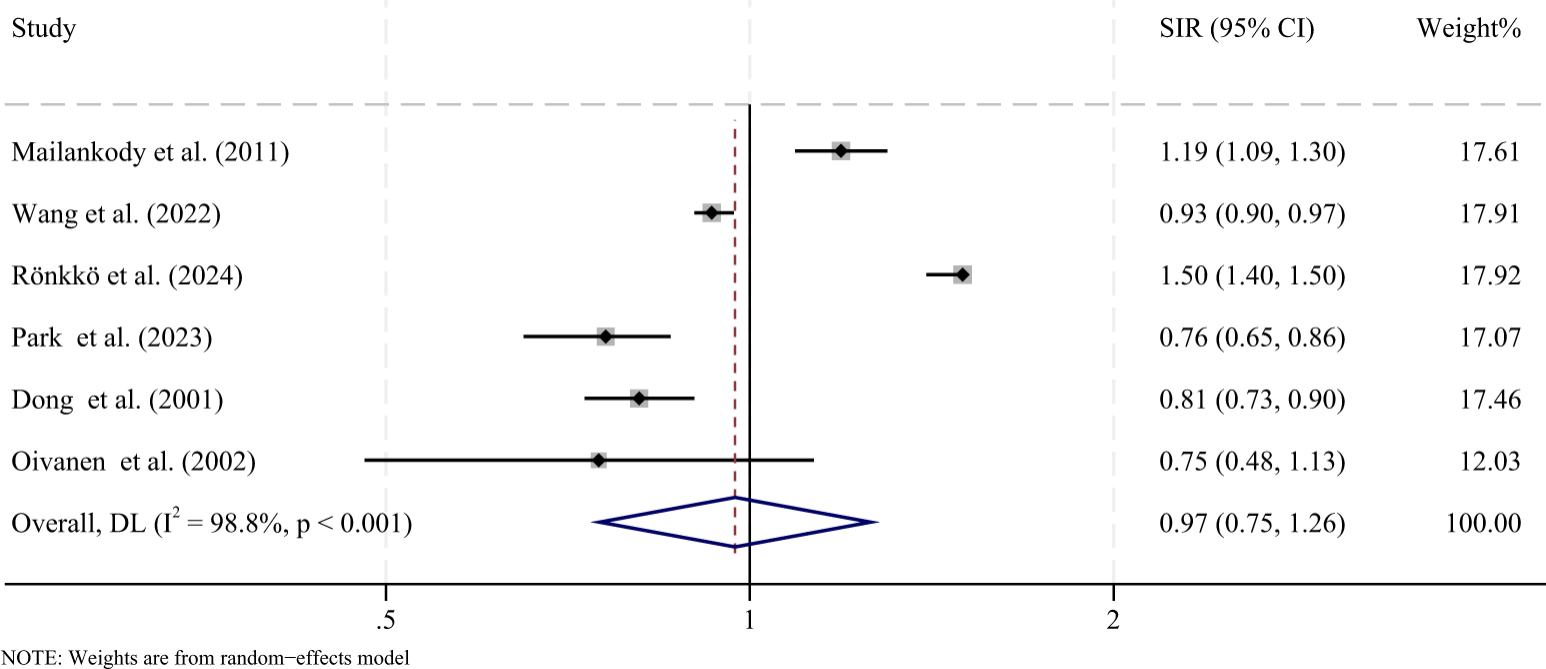
Forest plot showing the pooled standardized incidence ratios (SIRs) for second primary solid malignancies in patients with MM compared with the general population. CI, confidence interval; SIR, standardized incidence ratio; I2, inconsistency.

For solid tumors, six studies reported the overall risk. The pooled analysis found no significant difference in the risk of solid tumor SPMs between MM patients and the general population (SIR = 0.97, 95% CI: 0.75-1.26, P = 0.832) (**Figure 4**). Considerable heterogeneity was observed across these studies (I^2^ = 98.8%, P < 0.001). The result remained stable in sensitivity analyses (**Supplementary Figure 3**). Subgroup analyses were conducted to assess potential factors influencing the association between MM and overall second primary malignancies (**Table 2**). The results indicated no significant differences across the subgroups of year of diagnosis (before vs. after 2000), latency period (≤5 years vs. >5 years), or age (≤60 years vs. >60 years), sex (male vs. female).

### 3.6 Risk of site-specific second primary malignancies

**Supplementary Table 1** summarizes the meta-analysis results by cancer site. Among hematologic malignancies, patients with MM had higher risks for non-Hodgkin lymphoma (NHL) (SIR = 1.56, 95% CI: 1.12-2.19; P = 0.009), myelodysplastic syndromes (MDS) (SIR = 57.9, 95% CI: 25.08-133.67; P<0.001), and acute myeloid leukemia (AML) (SIR = 3.33, 95% CI: 2.14-5.20; P<0.001). The increase was most pronounced for MDS. In contrast, no significant differences were found for Hodgkin lymphoma, leukemia, lymphoid leukemia, or chronic myeloid leukemia (CML). A significantly lower risk was observed for chronic lymphocytic leukemia (CLL) (SIR = 0.18, 95% CI: 0.05-0.73; P = 0.017), although this finding was based on only two studies and should be interpreted with caution.

For solid tumors (**Supplementary Table 1**), patients with MM had significantly increased risks of mesothelioma (SIR = 1.68, 95% CI: 1.16-2.42; P = 0.006), skin cancer (SIR = 2.64, 95% CI: 1.80-3.85; P<0.001), melanoma (SIR = 1.57, 95% CI: 1.13-2.18; P = 0.008), endocrine cancers (SIR = 1.73, 95% CI: 1.08-2.79; P = 0.024), and thyroid cancer (SIR = 1.40, 95% CI: 1.01-1.95; P = 0.041). For most other solid tumors, including those of the oral cavity, pharynx, esophagus, stomach, colorectum, liver, pancreas, lung, kidney, reproductive system, and nervous system, no significant differences in risk were observed. However, breast cancer showed a significantly reduced risk (SIR = 0.69, 95% CI: 0.48-0.98; P = 0.04), a finding based on 9 studies. Risk reductions were also seen for head and neck cancer (SIR = 0.54) and tracheal/bronchial/lung cancer (SIR = 0.32), but these results were based on only two studies and should be interpreted cautiously. The reduction in bladder cancer risk was of borderline significance (SIR = 0.48, 95% CI: 0.23-1.00; P = 0.05).

### 3.7 Publication bias

We assessed publication bias in ten studies that reported overall SPM incidence in patients with MM compared with the general population. The funnel plot appeared approximately symmetrical (**Supplementary Figure 4**), suggesting minimal bias. Begg’s test (P = 0.592) and Egger’s test (P = 0.527) indicated no significant publication bias (**Supplementary Figures 5**, **6**).

## 4 Discussion

Advances in MM treatment have extended survival for many patients, but the emergence of a SPM remains a serious issue. Such diagnoses can influence not only clinical outcomes but also the patient’s emotional state (35–37). As survival improves, SPMs are appearing more often in long-term management, so clinicians have started to pay more attention to them when designing treatment plans (7). In this analysis, we tried to give a full picture of SPM risk in MM by comparing it directly with the general population. The overall risks, both overall SPMs and solid tumors—did not show a clear increase. However, hematologic SPMs were different, showing a marked rise. When looking at specific categories, the risks for NHL, MDS, and AML were higher, with MDS standing out as the most increased. For solid tumors, we still observed several elevated risks, particularly for mesothelioma, skin cancers, melanoma, endocrine system cancers, and thyroid cancer. The higher rate of skin cancer suggests that regular skin checks may be helpful. One unexpected finding was the lower risk of CLL in MM patients. Lower risks were also noted for cancers of the head and neck region, the trachea/bronchi/lungs, bladder cancer, and breast cancer.

The emergence of SPMs in MM patients is generally understood to result from several overlapping influences (7). For treatment-related causes, clinicians mainly focus on alkylating drugs like Melphalan and on IMiDs, where Lenalidomide is the central concern (7, 18, 58). High-dose Melphalan is still the standard conditioning regimen before ASCT in MM (3). The issue is that this approach carries a recognized long-term association with therapy-related myeloid neoplasms (t-MNs) (14). Melphalan’s main mechanism is DNA cross-linking. While this effect is useful for eliminating myeloma cells, it also harms normal hematopoietic stem and progenitor cells (14). The resulting genomic injury often produces complex patterns of chromosomal loss, such as deletions of chromosomes 5, 7, and 17, as well as 12p loss and monosomy 18 (50, 59, 60). IMiDs have changed the course of MM, and Lenalidomide in particular has contributed substantially to improved survival. At the same time, reports linking Lenalidomide to higher SPM risks have continued to increase (50, 61). One meta-analysis noted that Lenalidomide exposure increases both solid and hematologic SPMs, regardless of transplant status (18). Another analysis confirmed a clear association between Lenalidomide maintenance and SPM development (22). At the molecular level, Lenalidomide binds cereblon (CRBN), a component of an E3 ubiquitin ligase (62). This binding underlies its therapeutic activity but may also be involved in SPM biology (63). In B cells, Lenalidomide functions as a “molecular glue,” redirecting the CRBN complex to degrade Ikaros and Aiolos, which interferes with normal B-cell maturation (63, 64). In MDS, Lenalidomide can promote degradation of CK1α, a process that may particularly benefit the expansion of TP53-mutated hematopoietic stem and progenitor cells (63, 64). Effects on T-cell activity and the tumor microenvironment may further permit abnormal clonal growth (65, 66). Importantly, treatment interactions complicate interpretation. For example, one meta-analysis found that combining Lenalidomide with Melphalan yields a higher rate of hematologic SPMs than Melphalan alone (23). Importantly, treatment interactions complicate interpretation. For example, one meta-analysis found that combining Lenalidomide with Melphalan yields a higher rate of hematologic SPMs than Melphalan alone (7, 23, 67). Mechanistic work suggests that CRBN also plays a role in nucleotide excision repair, so Lenalidomide-related disruption of the CRBN/DDB1 complex could hinder the repair of Melphalan-induced DNA damage, further increasing susceptibility to SPMs (14, 66). As for ASCT itself, the available evidence is largely reassuring. Most studies indicate that ASCT alone does not substantially elevate SPM risk (68, 69). Reports showing increased AML/MDS risk after ASCT usually involve patients who had already received alkylator-based induction and were later treated with Lenalidomide maintenance (33, 70). Thus, the prevailing view is that the increased risk is more attributable to prior alkylator exposure and subsequent IMiD use rather than to the transplant conditioning regimen itself (14, 71). Additionally, chemotherapy can shape clonal evolution by applying selective pressure that allows resistant minor clones to persist. These clones may expand over time and eventually undergo malignant transformation, contributing to SPM formation (26, 72).

Beyond treatment-related exposures, MM itself has long been viewed as a potential contributor to SPM risk (7, 11, 73). This view has been reinforced by studies showing that precursor and early disease states may already carry excess myeloid risk. For example, patients with smoldering MM (SMM), despite having received no prior therapy, have been reported to show a higher incidence of MDS/AML than the general population (74). In addition, a German cohort with long-term follow-up found that the IgG subtype was the most common among MM patients who subsequently developed SPMs (75), and higher disease burden may also be relevant, as patients with ISS stage III have been reported to experience more SPMs than those with ISS stage I (26). At the same time, MM is associated with substantial immune dysfunction, including disrupted T-cell signaling, chronic inflammatory activation, cytokine imbalance, and abnormalities in both innate and adaptive immune compartments (76–79). These changes include altered T-cell subsets, reduced CD4^+^ and CD8^+^ counts, shifts in the Th1/Th2 balance, and functional defects involving dendritic cells, neutrophils, and NKT cells (76). Such immune and microenvironmental disturbances may create conditions that facilitate the persistence or evolution of abnormal hematopoietic clones. More recent studies further suggest that this vulnerability may, at least in part, reflect pre-existing clonal hematopoietic abnormalities. In newly diagnosed MM, MDS-associated phenotypic abnormalities and clonal hematopoiesis can already be detected before treatment, supporting the notion that abnormal hematopoiesis may precede therapy rather than arise exclusively as its consequence (80, 81). In this context, clonal hematopoiesis of indeterminate potential (CHIP), and possibly clonal cytopenia of undetermined significance (CCUS) when cytopenias are present, may represent an underlying biological substrate for later myeloid neoplasms (80, 82). Importantly, these abnormalities do not necessarily imply a shared clonal origin with MM. Rather, they may indicate a parallel or pre-existing clonal vulnerability that, in the setting of MM-related immune dysregulation, marrow microenvironmental disturbance, and cumulative therapeutic pressure, becomes more likely to persist or expand (82, 83). From this perspective, treatment exposure may act less as the sole initiating factor than as a promoter or selective pressure superimposed on pre-existing susceptibility (29). This framework may help explain why hematologic SPMs in MM, particularly MDS and AML, cannot be fully attributed to therapy alone but more likely arise from an interplay between disease biology, clonal hematopoietic susceptibility, marrow microenvironmental dysfunction, and cumulative treatment exposure.

Host-related factors, including both genetic and non-genetic components, are recognized contributors to SPM development in MM patients (7). Although subgroup analyses did not show statistically significant differences in SPM risk by age (≤60 years vs. >60 years) or sex, some patterns warrant attention.

Patients aged ≤60 years showed higher SIR values for overall SPMs as well as for hematologic and solid tumors when compared with older individuals. This pattern may indicate that younger patients with MM are more prone to developing SPMs. Several factors could explain this. Older patients have a shorter remaining lifespan, which reduces the time during which late-occurring malignancies might develop. Younger patients, by contrast, live longer and therefore have a broader period in which SPMs can be detected. In addition, intensive treatment is less often offered to older patients, whereas younger patients are more likely to receive alkylator-based therapies, which have well-documented mutagenic effects (84). Earlier studies have also shown that chemotherapy can increase the mutational load in normal hematopoietic cells in a way that resembles natural aging (8). High-dose regimens have been linked to an increased likelihood of solid tumor SPMs as well (69). Regarding sex, male patients exhibited a higher overall SIR for SPMs, while females showed higher SIRs for both hematologic and solid tumor categories. This implies that sex may influence SPM susceptibility in different ways depending on the tumor type. Although none of these differences reached statistical significance, they point toward possible age- and sex-related variations that warrant confirmation in larger cohorts and further exploration of the mechanisms involved.

We also assessed whether the calendar year at diagnosis and the latency period influenced the risk of SPMs. The subgroup analyses did not reveal statistically significant differences for either factor, but several tendencies were noticeable. Patients diagnosed after 2000 had higher SIRs for overall SPMs and for hematologic types, while their risk for solid tumors was somewhat lower than in those diagnosed earlier. A reasonable explanation is the shift in treatment approaches. Before 2000, alkylating agents were used more extensively and have been associated with an increased incidence of solid tumors (7). After 2000, newer agents such as proteasome inhibitors and IMiDs came into routine use and markedly improved survival. Longer survival, however, also means longer exposure to therapy, and this may gradually raise the likelihood of hematologic malignancies over time (73, 85). For latency, no significant differences were detected between patients followed for ≤5 years and those with more than 5 years of follow-up. Even so, SIRs tended to be higher in the group with >5 years of follow-up across all SPM categories. The pattern was most striking for hematologic SPMs: the risk was not elevated during the first few years but became clearly higher when follow-up extended beyond 5 years. This delayed increase is consistent with the idea that many SPMs take time to develop (9). These observations highlight the need for long-term surveillance, especially in younger patients or in those who have received several lines of therapy involving drugs with potential carcinogenic properties.

Although patients with MM showed higher risks for several SPM types, particularly hematologic malignancies, some cancers were reported less often than expected. A lower incidence was noted for CLL as well as for several solid tumors, including head and neck cancer, cancers of the trachea, bronchi and lung, bladder cancer, and breast cancer. The finding related to CLL is of particular interest. In contrast to the general increase observed for most hematologic SPMs, CLL showed a clear decline across two large population-based cohorts involving 69,290 MM patients, with both studies reporting the same direction of effect and no heterogeneity. This opposite pattern, observed despite higher risks for other hematologic malignancies, may indicate a relationship between MM and CLL that warrants cautious further study. For solid tumors, the reduced risks of head and neck cancer, respiratory tract cancers, bladder cancer, and breast cancer followed a similar trend. The estimate for tracheal/bronchial/lung cancer came from two studies that showed substantial heterogeneity, and when lung cancer was analyzed alone, the incidence did not differ significantly. This inconsistency suggests that variations in diagnosis or screening may contribute to the apparent decline in respiratory cancers. The lower rate of head and neck cancer also came from two studies; heterogeneity was absent, but the limited number of cases makes the result tentative. The decrease in bladder cancer risk was only marginal. Taken together, these findings may partially reflect differences in surveillance or diagnostic practice. Larger datasets will be needed to verify these observations. Although ALL was not included in the present quantitative synthesis because the currently available SIR-based studies were derived from overlapping SEER datasets and more recent clinically relevant reports did not provide SIR estimates, emerging evidence suggests that ALL remains a rare but clinically meaningful hematologic SPM in selected MM patients, particularly after ASCT followed by lenalidomide maintenance. Reported cases appear to be predominantly Philadelphia chromosome-negative and may have a relatively favorable outcome after treatment, indicating that ALL arising after MM may differ clinically from the more commonly recognized therapy-related myeloid neoplasms (27–32).

Although some solid tumors appeared to occur less often than expected, these findings should be interpreted cautiously. In our analysis, lower incidences were observed for head and neck cancer, tracheal/bronchial/lung cancer, bladder cancer, and breast cancer. However, several of these estimates were derived from only a small number of studies and may have been affected by heterogeneity, differences in surveillance, or incomplete ascertainment. A lower incidence of breast cancer was observed; however, this finding warrants cautious interpretation due to potential competing risk and screening effects, and requires further investigation (54, 69, 86–88). Data from individual cohorts also suggest that site-specific solid tumor patterns may vary across clinical settings. For example, the US Connect MM Registry, which included approximately 3,000 patients with newly diagnosed MM, reported a higher incidence of certain solid tumors, including lung and prostate cancer. This finding suggests that second malignancy patterns may differ according to patient population, treatment exposure, and follow-up duration, and therefore reduced risks for individual solid tumors in pooled analyses should be interpreted cautiously (89).

One strength of the present study is that it synthesized standardized incidence ratio (SIR)-based evidence, allowing direct comparison between the observed incidence of SPMs in MM patients and that expected in the general population. Unlike treatment-specific meta-analyses, this approach captures the overall excess burden of SPMs at the population level. This distinction is particularly important in MM, where patients are often exposed to multiple sequential or combined therapies over the course of their disease, making treatment-specific estimates insufficient to fully reflect long-term second malignancy risk (18, 21–23, 61). Therefore, our findings complement prior treatment-focused analyses by providing a population-level perspective on the overall burden of SPMs in patients with MM. Several limitations should be acknowledged. First, substantial heterogeneity was present across several pooled analyses, and the summary SIR estimates should therefore be interpreted with caution. This likely reflects, at least in part, the long time span covered by the included studies, from the 1970s to the 2020s, during which MM treatment changed markedly. Although we performed a subgroup analysis using the year 2000 as a cutoff, heterogeneity remained high, probably because the treatment eras were still too broadly grouped. More refined stratification was not possible because of the limited number of studies and incomplete reporting. In addition, the studies came from different geographic regions and cancer registries, which may also have contributed to variation in case ascertainment, background cancer incidence, and follow-up practice. The original reports often lacked standardized information on treatment, MM subtype, and disease stage, which further limited more detailed subgroup analyses. Although we examined diagnostic period, age, and latency, narrower age bands and more specific latency intervals could not be assessed.

Future changes in MM treatment may also affect long-term SPM risk, although this remains uncertain. Approaches that reduce cumulative alkylator exposure, shorten maintenance, or rely more on newer immunotherapies may eventually alter second malignancy patterns, but current evidence is not sufficient to show that they reduce SPM incidence, and longer follow-up in contemporary cohorts is needed (39, 90, 91). From a clinical perspective, SPM risk should be discussed with patients in clear and balanced terms. Follow-up should include review of blood counts, attention to suspicious imaging findings, and continuation of age-appropriate cancer screening; when clinically indicated, further evaluation such as targeted imaging, dermatologic assessment, or bone marrow examination may be warranted. This is particularly relevant because incidental imaging abnormalities can occasionally reveal clinically significant second malignancies that require timely investigation and coordinated management (39, 92).

## 5 Conclusion

In summary, this meta-analysis indicates that patients with MM have a higher risk of hematologic SPMs than the general population. The increase is most evident for NHL, MDS, AML, mesothelioma, skin cancer, melanoma, endocrine tumors, and thyroid cancer. In contrast, CLL and breast cancer occurred less often than expected. These findings underscore the distinctive SPM profile in patients with MM and support the need for tumor-specific surveillance strategies. Monitoring should place particular emphasis on hematologic SPMs, as well as on key solid tumors such as skin cancer, melanoma, endocrine malignancies, thyroid cancer, and mesothelioma. Further studies are warranted to clarify the mechanisms underlying these site-specific increases. The reduced incidence of breast cancer in patients with also merits attention, and prospective research is needed to confirm this pattern and explore potential explanations. Overall, follow-up care should adopt a differentiated, tumor-specific approach rather than a uniform strategy for all SPMs.

## Data Availability

The original contributions presented in the study are included in the article/**Supplementary Material**. Further inquiries can be directed to the corresponding author/s.
