# Supplementary material for: Risk for second primary malignancies in patients with multiple myeloma: a systematic review and meta-analysis
Source: Front Oncol. 2026 Apr 7;16:1770120. doi: 10.3389/fonc.2026.1770120 (PMC13095524; doi:10.3389/fonc.2026.1770120)
Supplement: Supplementary file 1 [file DataSheet1.docx]

**Risk for second primary malignancies in Multiple Myeloma survivors: a systematic review and meta-analysis**

**“Online Supplementary Material”**

Supplemental Appendix 1:

Full search strategy (August 15, 2025): PubMed

| **Searches** |
| --- |
| #1 (("Multiple Myeloma"[Mesh] OR "Smoldering Multiple Myeloma"[Mesh]) OR "Monoclonal Gammopathy of Undetermined Significance"[Mesh]) OR (((((((((Multiple Myeloma[Title/Abstract]) OR (Smoldering Multiple Myeloma[Title/Abstract])) OR (Monoclonal Gammopathy of Undetermined Significance[Title/Abstract])) OR (Plasma Cell Myeloma[Title/Abstract])) OR (Myelomas, Multiple[Title/Abstract])) OR (Myeloma, Plasma-Cell[Title/Abstract])) OR (Cell Myeloma, Plasma[Title/Abstract])) OR (Kahler Disease[Title/Abstract])) OR (Myelomatosis[Title/Abstract]))  #2 ("Neoplasms, Second Primary"[Mesh]) OR ((((((((((((((((((((((((((((((((((Neoplasm, Second Primary[Title/Abstract]) OR (Second Primary Neoplasm[Title/Abstract])) OR (Neoplasms, Metachronous[Title/Abstract])) OR (Neoplasms, Metachronous Second Primary[Title/Abstract])) OR (Second Malignancy[Title/Abstract])) OR (Malignancy, Second[Title/Abstract])) OR (Second Neoplasm[Title/Abstract])) OR (Neoplasm, Second[Title/Abstract])) OR (Second Primary Neoplasms, Metachronous[Title/Abstract])) OR (Metachronous Second Primary Neoplasms[Title/Abstract])) OR (Metachronous Neoplasms[Title/Abstract])) OR (Neoplasms, Therapy-Associated[Title/Abstract])) OR (Therapy-Associated Neoplasm[Title/Abstract])) OR (Neoplasm, Therapy-Related[Title/Abstract])) OR (Therapy-Related Neoplasm[Title/Abstract])) OR (Neoplasms, Treatment-Associated[Title/Abstract])) OR (Treatment-Associated Neoplasm[Title/Abstract])) OR (Neoplasms, Treatment Related[Title/Abstract])) OR (Treatment-Related Neoplasm[Title/Abstract])) OR (Cancer, Second Primary[Title/Abstract])) OR (Second Primary Cancer[Title/Abstract])) OR (Second Cancer[Title/Abstract])) OR (Cancer, Second[Title/Abstract])) OR (Therapy-Associated Cancer[Title/Abstract])) OR (Cancer, Therapy-Associated[Title/Abstract])) OR (Therapy Associated Cancer[Title/Abstract])) OR (Cancer, Treatment-Associated[Title/Abstract])) OR (Treatment Associated Cancer[Title/Abstract])) OR (Therapy-Related Cancer[Title/Abstract])) OR (Cancer, Therapy-Related[Title/Abstract])) OR (Therapy Related Cancer[Title/Abstract])) OR (Treatment-Related Cancer[Title/Abstract])) OR (Cancer, Treatment-Related[Title/Abstract])) OR (Treatment Related Cancer[Title/Abstract]))  #3 #1 AND #2  #4 "animals"[MeSH Terms] NOT "humans"[MeSH Terms]  #5 #3 NOT #4  #6 limit #5 to English language |

Supplemental Appendix 2: Full-text publications excluded with reason

| **Online Supplementary Reference** | **Reason for exclusion** |
| --- | --- |
| (1-10) | Inappropriate study population (n=10) |
| (11) | Inappropriate study type (n=1) |
| (12-33) | Inappropriate outcome (n=22) |
| (34-36) | Overlapping population (n=3) |
| (37) | Unretrievable original literature (n=1) |

**Reference**

1. Okines A, Thomson CS, Radstone CR, Horsman JM, Hancock BW. Second primary malignancies after treatment for malignant lymphoma. British Journal of Cancer. 2005;93(4):418-24.

2. Barzenje DA, Kolstad A, Ghanima W, Holte H. Long-term outcome of patients with solitary plasmacytoma treated with radiotherapy: A population-based, single-center study with median follow-up of 13.7 years. Hematological Oncology. 2018;36(1):217-23.

3. Varkonyi J, Kovalszky I, Nemeth A, Demeter J, Raposa T. Increased risk for cancer in multiple myeloma patients and their first-degree relatives. Haematologia. 2001;31(1):45-50.

4. Tzeng HE, Lin CL, Tsai CH, Tang CH, Hwang WL, Cheng YW, et al. Time trend of multiple myeloma and associated secondary primary malignancies in Asian patients: a Taiwan population-based study. PloS one. 2013;8(7):e68041.

5. Storm HH, Prener A. Second cancer following lymphatic and hematopoietic cancers in Denmark, 1943-80. National Cancer Institute monograph. 1985;68:389-409.

6. Arora M, Chen Y, Hageman L, Wu J, Landier W, Francisco L, et al. Morbidity burden in survivors of multiple myeloma who underwent autologous transplantation: A Bone Marrow Transplantation Survivor Study. Cancer. 2020;126(14):3322-9.

7. Robinson AA, Wang J, Vardanyan S, Madden EK, Hebroni F, Udd KA, et al. Risk of skin cancer in multiple myeloma patients: a retrospective cohort study. European journal of haematology. 2016;97(5):439-44.

8. Ojha RP, Evans EL, Felini MJ, Singh KP, Thertulien R. The association between renal cell carcinoma and multiple myeloma: insights from population-based data. BJU international. 2011;108(6):825-30.

9. Howard RA, Dores GM, Curtis RE, Anderson WF, Travis LB. Merkel cell carcinoma and multiple primary cancers. Cancer epidemiology, biomarkers & prevention : a publication of the American Association for Cancer Research, cosponsored by the American Society of Preventive Oncology. 2006;15(8):1545-9.

10. El-Fattah MA. Second cancers in survivors of plasmacytoma of bone in the U.S.A: a SEER database analysis. Leukemia and Lymphoma. 2017;58(12):2952-6.

11. Miguel JFS, Schlag R, Khuageva NK, Dimopoulos MA, Shpilberg O, Kropff M, et al. Persistent overall survival benefit and no increased risk of second malignancies with bortezomib-melphalan-prednisone versus melphalan-prednisone in patients with previously untreated multiple myeloma. Journal of Clinical Oncology. 2013;31(4):448-55.

12. Munker R, Baghian A, Koleva Y, Andrews P, Matharoo GS, Wright AE, et al. Long-term follow-up of patients with multiple myeloma treated with total body irradiation—Melphalan conditioning. European Journal of Haematology. 2017;99(1):56-9.

13. Munker R, Shi R, Lin D, Guo S, Hayes TG. Multiple myeloma and other malignancies: a pilot study from the Houston VA. Clinical lymphoma, myeloma & leukemia. 2014;14(2):102-6.

14. Patel KK, Shah JJ, Feng L, Lee HC, Manasanch EM, Olsem J, et al. Safety and Efficacy of Combination Maintenance Therapy with Ixazomib and Lenalidomide in Patients with Posttransplant Myeloma. Clinical cancer research : an official journal of the American Association for Cancer Research. 2022;28(7):1277-84.

15. Kobayashi Y, Arimoto H, Watanabe S. Second malignant lymphomas and leukemias in the National Cancer Center from 1962 to 1987. Japanese Journal of Cancer Research. 1990;81(6-7):570-7.

16. Grudeva-Popova J, Nenova I, Spasova M, Yaneva M, Beleva E, Ananoshtev N. Multiple myeloma in association with second malignancy. Journal of BUON : official journal of the Balkan Union of Oncology. 2013;18(2):448-52.

17. Cooper JD, Shou K, Sunderland K, Pham K, Thornton JA, DeStefano CB. Real-World Pitfalls of Analyzing Real-World Data: A Cautionary Note and Path Forward. JCO clinical cancer informatics. 2023;7:e2300097.

18. Krishnan AY, Mei M, Sun CL, Thomas SH, Teh JB, Kang T, et al. Second primary malignancies after autologous hematopoietic cell transplantation for multiple myeloma. Biology of blood and marrow transplantation : journal of the American Society for Blood and Marrow Transplantation. 2013;19(2):260-5.

19. Ormerod A, Fausel CA, Abonour R, Kiel PJ. Observations of second primary malignancy in patients with multiple myeloma. Clinical lymphoma, myeloma & leukemia. 2012;12(2):113-7.

20. Kotchetkov R, Masih-Khan E, Chu CM, Atenafu EG, Chen C, Kukreti V, et al. Secondary primary malignancies during the lenalidomide–dexamethasone regimen in relapsed/refractory multiple myeloma patients. Cancer Medicine. 2017;6(1):3-11.

21. Fei F, Reddy V, Rosenblum F. Secondary primary malignancies in patients with multiple myeloma: A single institution experience. Hematological oncology. 2021;39(5):674-9.

22. Liu Y, Hou HA, Qiu H, Tang CH. Is the risk of second primary malignancy increased in multiple myeloma in the novel therapy era? A population-based, retrospective cohort study in Taiwan. Scientific reports. 2020;10(1):14393.

23. Miles B, Mackey JD. Increased Risk of Second Primary Malignancy and Mortality at ten Years After Stem Cell Transplant for Multiple Myeloma: An Analysis of 14,532 Patients. Cureus. 2021;13(7):e16372.

24. Rosenberg AS, Brunson A, Tuscano J, Jonas BA, Hoeg R, Wun T, et al. Effect of autologous hematopoietic stem cell transplant on the development of second primary malignancies in multiple myeloma patients. Blood cancer journal. 2021;11(1):5.

25. Rollison DE, Komrokji R, Lee JH, Hampras S, Fulp W, Fisher K, et al. Subsequent primary malignancies among multiple myeloma patients treated with or without lenalidomide. Leukemia & lymphoma. 2017;58(3):560-8.

26. Jones JR, Cairns DA, Gregory WM, Collett C, Pawlyn C, Sigsworth R, et al. Second malignancies in the context of lenalidomide treatment: an analysis of 2732 myeloma patients enrolled to the Myeloma XI trial. Blood cancer journal. 2016;6(12):e506.

27. Ko H, Han S, Park SS, Choi S, Byun JM, Min CK. Risk of Secondary Malignancies After Multiple Myeloma: A Nationwide Case-Control Cohort Study. Clinical lymphoma, myeloma & leukemia. 2024;24(10):e366-e75.

28. Brink M, Minnema MC, Visser O, Levin MD, Posthuma E, Broijl A, et al. Increased mortality risk in multiple-myeloma patients with subsequent malignancies: a population-based study in the Netherlands. Blood cancer journal. 2022;12(3):41.

29. Barth P, Castillo JJ, Olszewski AJ. Outcomes of secondary solid tumor malignancies among patients with myeloma: A population-based study. Cancer. 2019;125(4):550-8.

30. Ailawadhi S, Swaika A, Razavi P, Yang D, Chanan-Khan A. Variable risk of second primary malignancy in multiple myeloma patients of different ethnic subgroups. Blood cancer journal. 2014;4(9):e243.

31. Gibson S, Thornton J, Sunderland K, Pham K, DeStefano C. Multiple Myeloma in Adolescent and Young Adults: An ASCO CancerLinQ and SEER Analysis. Clinical Lymphoma, Myeloma and Leukemia. 2023;23(10):e335-e40.

32. Eisfeld C, Kajüter H, Möller L, Wellmann I, Shumilov E, Stang A. Time trends in survival and causes of death in multiple myeloma: a population-based study from Germany. BMC cancer. 2023;23(1):317.

33. Engelhardt M, Ihorst G, Landgren O, Pantic M, Reinhardt H, Waldschmidt J, et al. Large registry analysis to accurately define second malignancy rates and risks in a well-characterized cohort of 744 consecutive multiple myeloma patients followed-up for 25 years. Haematologica. 2015;100(10):1340-9.

34. Chen T, Fallah M, Jansen L, Castro FA, Krilavicuite A, Katalinic A, et al. Distribution and risk of the second discordant primary cancers combined after a specific first primary cancer in German and Swedish cancer registries. Cancer Letters. 2015;369(1):152-66.

35. Costa LJ, Godby KN, Chhabra S, Cornell RF, Hari P, Bhatia S. Second primary malignancy after multiple myeloma-population trends and cause-specific mortality. British journal of haematology. 2018;182(4):513-20.

36. Razavi P, Rand KA, Cozen W, Chanan-Khan A, Usmani S, Ailawadhi S. Patterns of second primary malignancy risk in multiple myeloma patients before and after the introduction of novel therapeutics. Blood cancer journal. 2013;3(6):e121.

37. Boice JD, Jr., Curtis RE, Kleinerman RA, Flannery JT, Fraumeni JF, Jr. Multiple primary cancers in Connecticut, 1935-82. The Yale journal of biology and medicine. 1986;59(5):533-45.

**
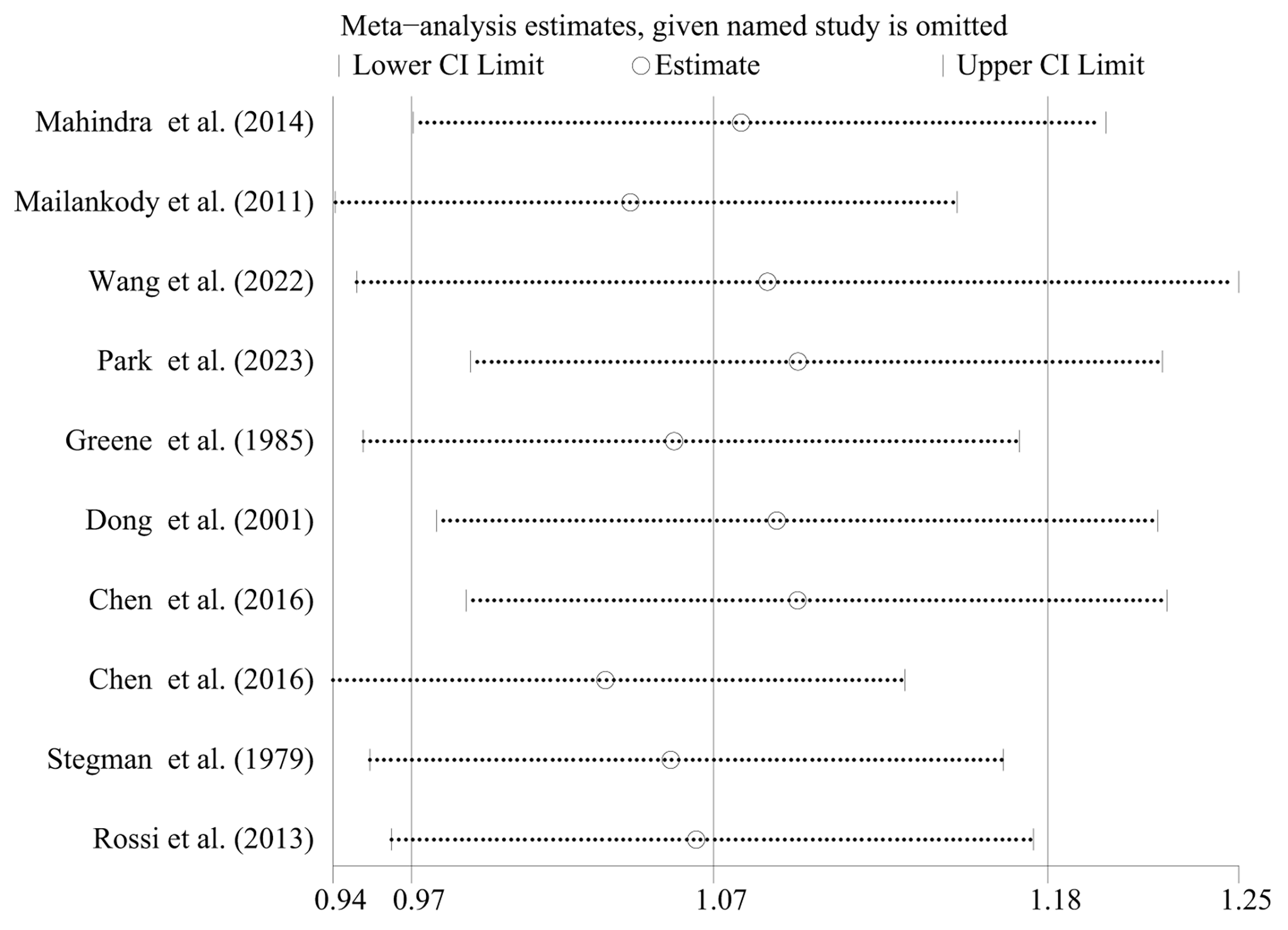
**

**Supplemental Figure 1.** Sensitivity analysis for the meta-analysis evaluating the risk of overall second primary malignancies in patients with multiple myeloma (MM) compared with the general population (SIR). The pooled standardized incidence ratios and their 95% CI were recalculated after removing each study one at a time. CI, confidence interval; SIR, standardized incidence ratio.


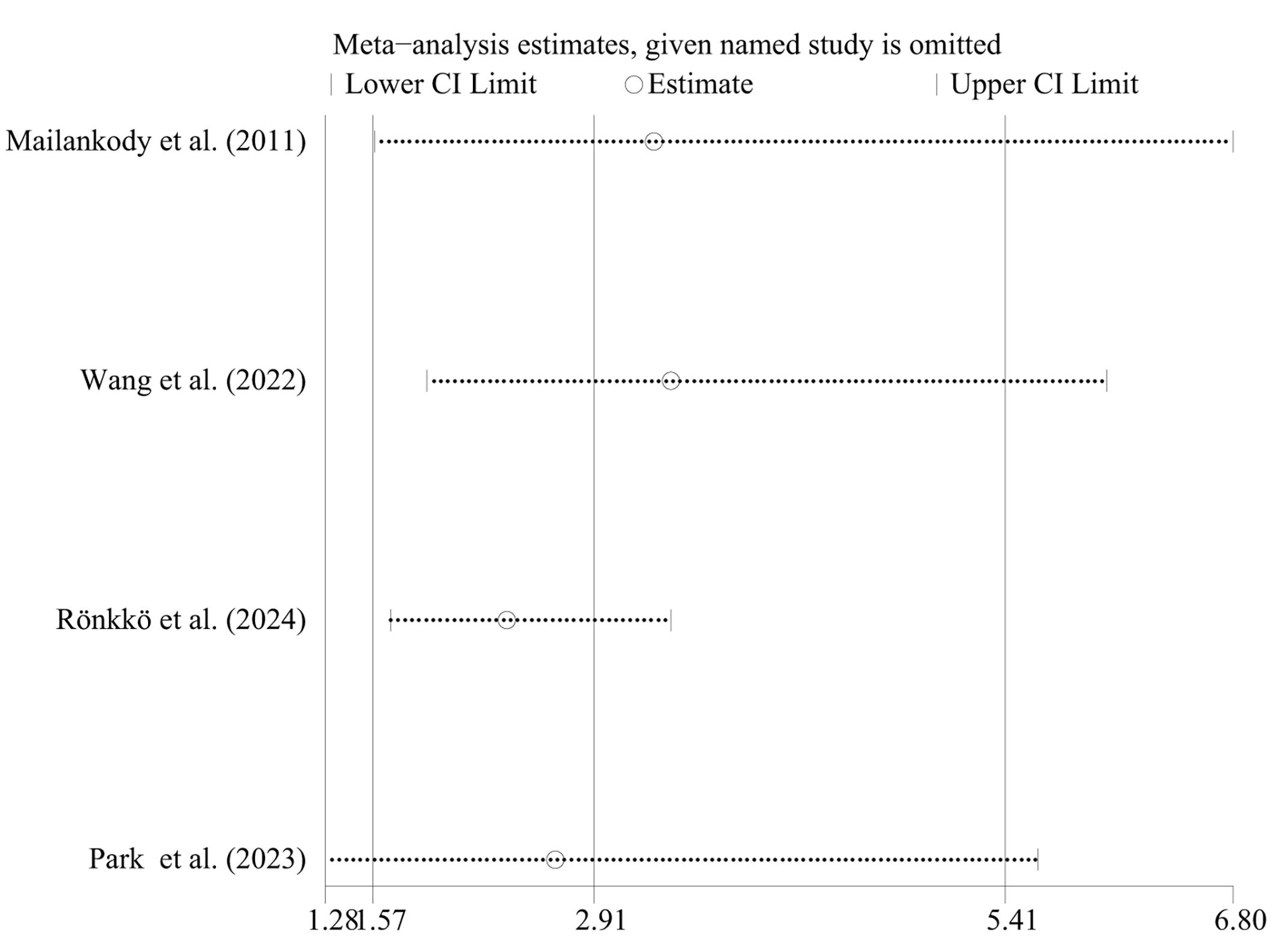


**Supplemental Figure 2.** Sensitivity analysis for the meta-analysis assessing the risk of second primary hematologic malignancies in patients with multiple myeloma (MM) compared with the general population (SIR). The pooled standardized incidence ratios and their 95% confidence intervals were recomputed after excluding each study one at a time. CI, confidence interval; SIR, standardized incidence ratio.


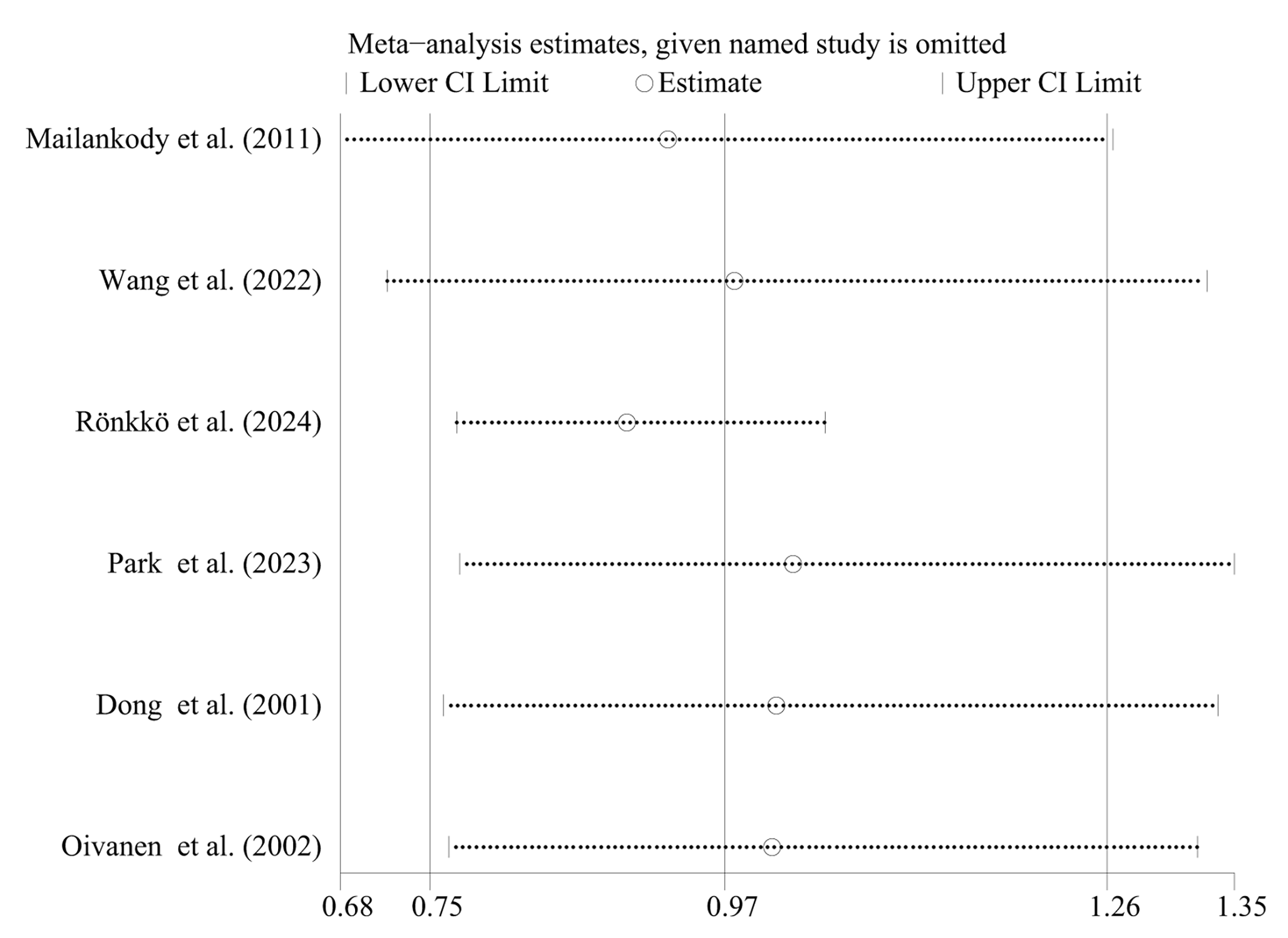


**Supplemental Figure 3.** Sensitivity analysis for the meta-analysis examining the risk of second primary solid malignancies in individuals with multiple myeloma (MM) compared with the general population (SIR). For this analysis, pooled standardized incidence ratios and 95% confidence intervals were recalculated sequentially, omitting one study at each step. CI, confidence interval; SIR, standardized incidence ratio.

**
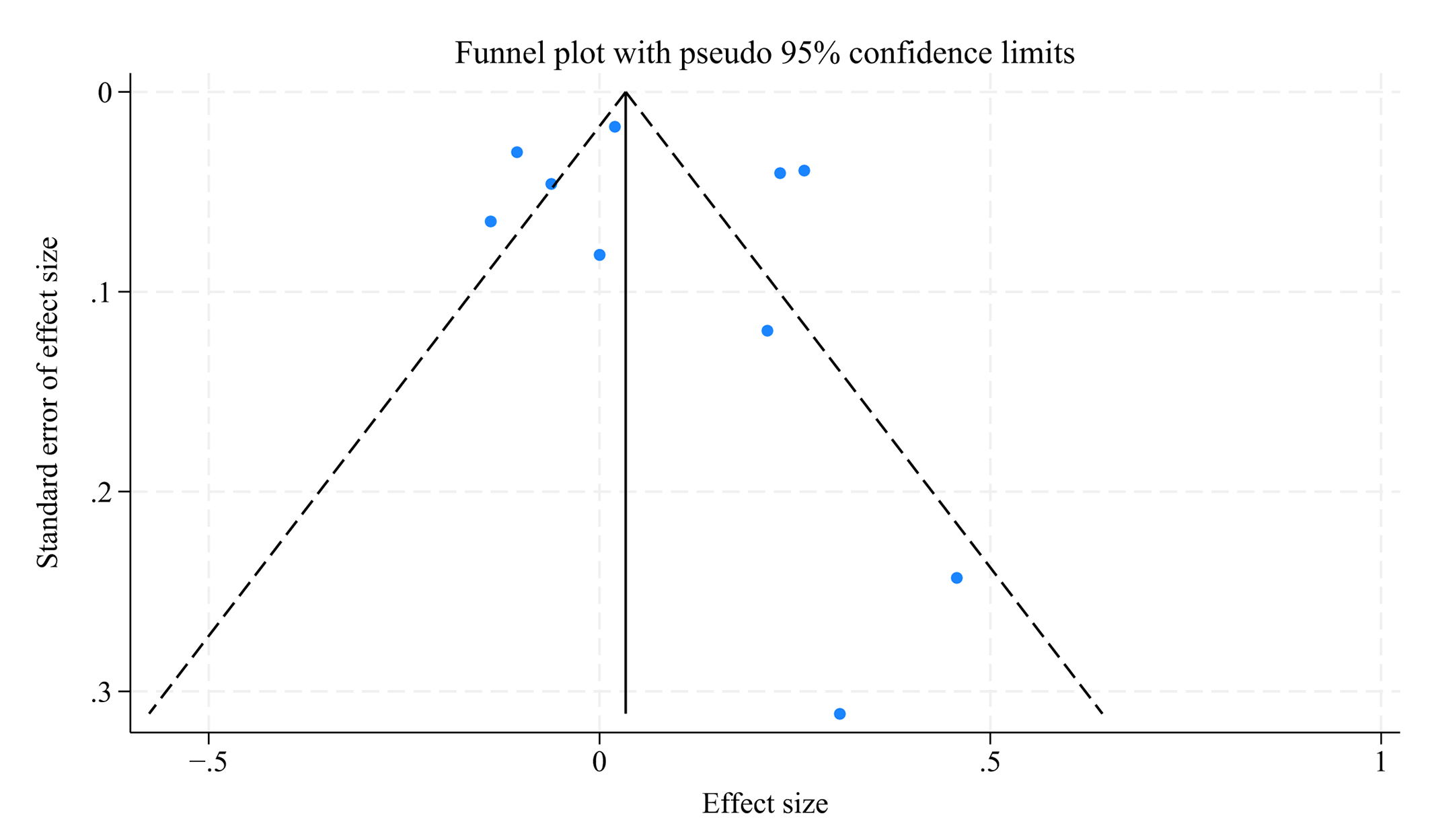
**

**Supplemental Figure 4.** Funnel plot illustrating the distribution of studies included in the analysis of overall second primary malignancies in multiple myeloma (MM) survivors compared with the general population (SIR).


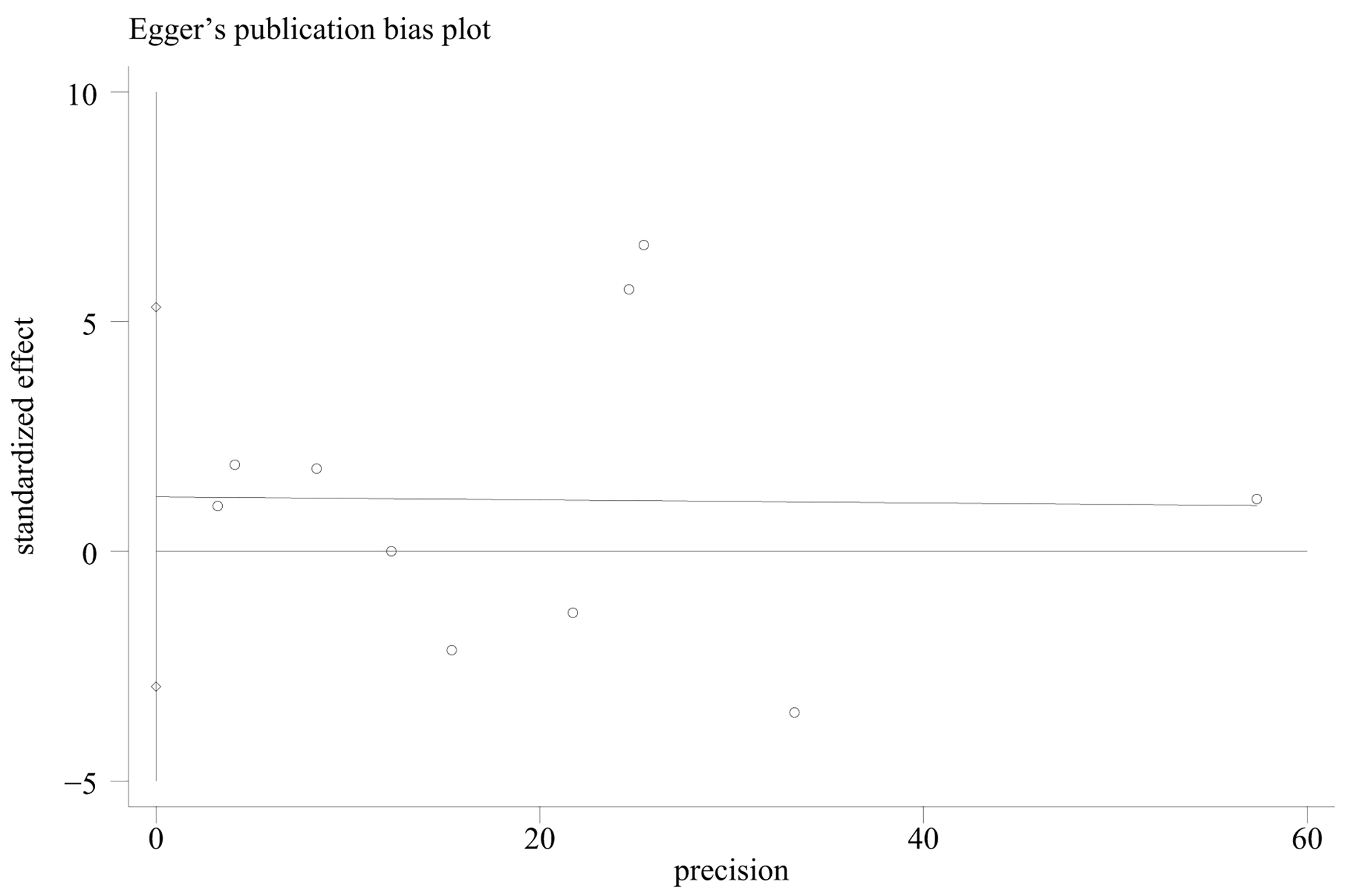


**Supplemental Figure 5.** Egger’s test used to assess potential publication bias in the meta-analysis evaluating overall second primary malignancies among survivors of multiple myeloma (MM).


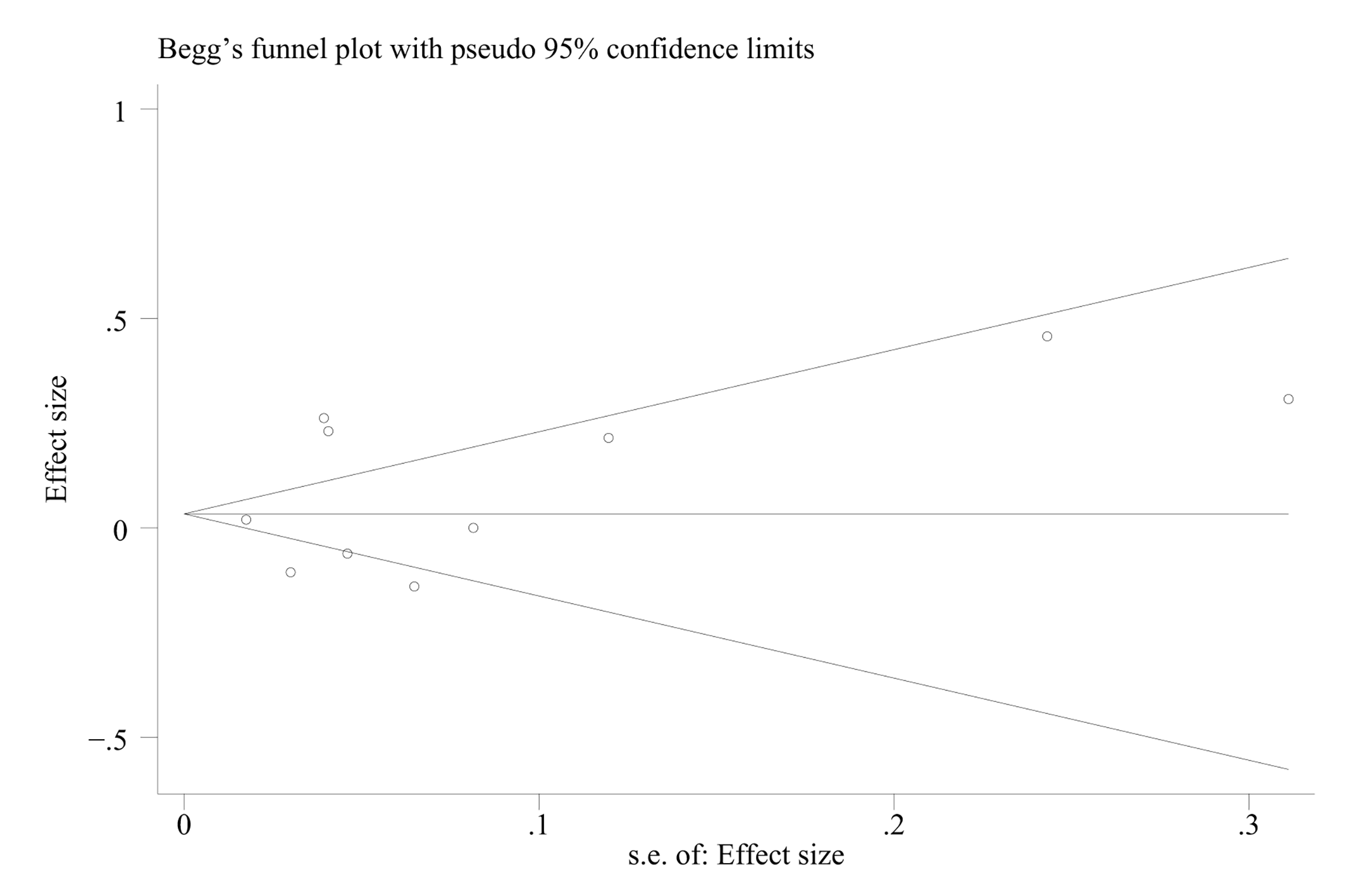


**Supplemental Figure 6.** Begg’s test examining publication bias in the meta-analysis of overall second primary malignancies in individuals with multiple myeloma (MM) compared with the general population.

**Supplemental Table 1.** Site-specific meta-analysis of second primary malignancy (SPMs) risks in survivors of multiple myeloma (MM) compared with cancer rates in the general population.

| Second primary malignancy | | N | SIR (95%CI) | P Value | I2 (%) | Pheterogeneity Value |
| --- | --- | --- | --- | --- | --- | --- |
| **Hematologic malignancies** | | | | | | |
|  | Lymphoma | 2 | 2.27 (0.96-5.36) | 0.062 | 93.700 | ＜0.001 |
|  | HL | 4 | 2.10 (0.82-5.39) | 0.123 | 0.000 | 0.51 |
|  | NHL | 7 | 1.56 (1.12-2.19) | 0.009 | 70.500 | 0.002 |
|  | MDS | 2 | 57.90 (25.08-133.67) | ＜0.001 | 79.900 | 0.03 |
|  | Leukemia | 3 | 2.65 (0.89-7.89) | 0.079 | 97.700 | ＜0.001 |
|  | Lymphoid leukemia | 3 | 1.41 (0.75-2.66) | 0.285 | 30.800 | 0.24 |
|  | CLL | 2 | 0.18 (0.05-0.73) | 0.017 | 0.000 | 0.89 |
|  | AML | 4 | 3.33 (2.14-5.20) | ＜0.001 | 71.300 | 0.02 |
|  | CML | 2 | 1.29 (0.44-3.77) | 0.645 | 0.000 | 0.62 |
| **Solid tumors** | | | | | | |
|  | Head and neck cancer | 2 | 0.54 (0.30-0.98) | 0.041 | 0.000 | 0.92 |
|  | Oral cavity and pharyngeal cancer | 2 | 0.84 (0.12-5.61) | 0.853 | 94.400 | ＜0.001 |
|  | Esophageal cancer | 2 | 0.52 (0.07-3.78) | 0.515 | 84.600 | 0.01 |
|  | Stomach cancer | 4 | 0.88 (0.45-1.72) | 0.712 | 74.600 | 0.01 |
|  | Colorectal cancer | 4 | 0.98 (0.73-1.30) | 0.870 | 81.800 | 0.00 |
|  | Colon cancer | 3 | 0.47 (0.13-1.76) | 0.264 | 85.400 | 0.00 |
|  | Rectal cancer | 3 | 0.57 (0.11-2.91) | 0.501 | 74.100 | 0.02 |
|  | Hepatobiliary cancer | 4 | 0.96 (0.80-1.15) | 0.650 | 0.000 | 0.55 |
|  | Liver cancer | 3 | 0.60 (0.24-1.50) | 0.273 | 30.100 | 0.24 |
|  | Pancreatic cancer | 3 | 0.57 (0.15-2.13) | 0.403 | 75.500 | 0.02 |
|  | Respiratory system cancers | 3 | 0.58 (0.13-2.53) | 0.468 | 99.200 | ＜0.001 |
|  | Tracheal/Bronchial/Lung cancer | 2 | 0.32 (0.12-0.86) | 0.024 | 90.700 | 0.00 |
|  | Lung cancer | 4 | 0.94 (0.72-1.22) | 0.630 | 14.200 | 0.32 |
|  | Mesothelioma | 2 | 1.68 (1.16-2.42) | 0.006 | 0.000 | 0.90 |
|  | Kidney cancer | 7 | 1.45 (0.89-2.37) | 0.136 | 80.500 | ＜0.001 |
|  | Ureteral cancer | 2 | 1.92 (0.39-9.32) | 0.420 | 26.800 | 0.24 |
|  | Bladder cancer | 5 | 0.48 (0.23-1.00) | 0.050 | 74.700 | 0.00 |
|  | Breast cancer | 9 | 0.69 (0.48-0.98) | 0.040 | 85.800 | ＜0.001 |
|  | Female reproductive system cancers | 4 | 0.61 (0.28-1.34) | 0.218 | 87.800 | ＜0.001 |
|  | Ovarian cancer | 3 | 0.51 (0.15-1.70) | 0.269 | 42.200 | 0.18 |
|  | Male reproductive system cancers | 3 | 0.70 (0.42-1.15) | 0.162 | 94.500 | ＜0.001 |
|  | Prostate cancer | 5 | 0.72 (0.43-1.19) | 0.194 | 89.200 | ＜0.001 |
|  | Nervous system cancers | 8 | 1.24 (0.86-1.77) | 0.246 | 60.900 | 0.01 |
|  | Skin cancer | 2 | 2.64 (1.80-3.85) | ＜0.001 | 80.900 | 0.022 |
|  | Melanoma | 6 | 1.57 (1.13-2.18) | 0.008 | 70.700 | 0.00 |
|  | Soft tissue | 4 | 1.65 (0.88-3.11) | 0.118 | 65.600 | 0.03 |
|  | Endocrine cancers | 3 | 1.73 (1.08-2.79) | 0.024 | 66.400 | 0.05 |
|  | Thyroid cancer | 2 | 1.40 (1.01-1.95) | 0.041 | 0.000 | 0.59 |

HL, Hodgkin lymphoma; NHL, Non-Hodgkin lymphoma ; MDS, Myelodysplastic syndromes MDS; CLL, Chronic lymphocytic leukemia; AML, Acute myeloid leukemia; CML, Chronic myeloid leukemia. 95% CI: 95% Confidence interval; N: Number of cohorts; SIR: Standardized incidence ratio
